# The Response of Farmland Bird Communities to Agricultural Intensity as Influenced by Its Spatial Aggregation

**DOI:** 10.1371/journal.pone.0119674

**Published:** 2015-03-23

**Authors:** Félix Teillard, Frédéric Jiguet, Muriel Tichit

**Affiliations:** 1 INRA-AgroParisTech, UMR 1048 SAD APT, Paris, France; 2 MNHN-CNRS-UPMC, UMR 7204 CRBPO, Paris, France; Università degli Studi di Napoli Federico II, ITALY

## Abstract

The shape of the relationship between biodiversity and agricultural intensity determines the range of intensities that should be targeted by conservation policies to obtain the greatest environmental benefits. Although preliminary evidence of this relationship exists, the influence of the spatial arrangement of intensity on biodiversity remains untested. We conducted a nationwide study linking agricultural intensity and its spatial arrangement to a farmland bird community of 22 species. Intensity was described with a continuous indicator based on Input Cost per hectare, which was relevant for both livestock and crop production. We used the French Breeding Bird Survey to compute several descriptors of the farmland bird community along the intensity gradient and tested for the significance of an interaction effect between intensity and its spatial aggregation on these descriptors. We found that the bird community was comprised of both winner and loser species with regard to intensity. The community composition descriptors (trophic level, specialisation, and specialisation for grassland indices) displayed non-linear relationships to intensity, with steeper slopes in the lower intensity range. We found a significant interaction effect between intensity and its spatial aggregation on the grassland specialisation index of the bird community; the effect of agricultural intensity was strengthened by its spatial aggregation. We suggest that an opportunity to improve the effectiveness of conservation policies exists by targeting measures in areas where intensity is moderate to low and aggregated. The effect of the aggregation of agricultural intensity on biodiversity should be considered in other scales and taxa when developing optimal policy targeting and intensity allocation strategies.

## Introduction

The decline in farmland biodiversity related to agricultural intensification [1–3] highlights the need to develop public policy aimed at reversing this trend and, more immediately, to improve the effectiveness of existing plans [4, 5]. To do so, the shape of the relationship between biodiversity and agricultural intensity should be quantified, which would help identify the range of intensities with the greatest environmental benefit [6]. Two main shapes of this relationship have been hypothesised: a convex shape, where biodiversity loss is greater when intensifying unfarmed and extensive habitats; and a concave shape, where biodiversity loss is greater at the highest intensities. Although some knowledge about the shape of the biodiversity/intensity relationship exists [7–9], evidence of differences due to species traits and the spatial arrangement of agricultural intensity are lacking.

The relationship between biodiversity and agricultural intensity varies by species group. Specialist species can be more sensitive to intensity than generalist species (for butterflies [10]; for birds [11]). Among farmland specialists, differences are also reported between grassland and non-grassland species [12, 13]. This distinction is particularly important in Europe, where grassland agro-ecosystems hold numerous steppic species adapted to open, extensive habitats [14, 15]. Functional traits of species can also influence their response to intensity, the trophic level in particular [16, 17]. Differences among species, therefore, need to be considered when computing biodiversity responses to intensity at the community level.

The spatial arrangement of agricultural intensity affects biodiversity. The intensity of the surrounding agricultural matrix affects species that use patches of semi-natural habitat [18, 19]. Devictor and Jiguet (2007) [20] further demonstrated the effects of various surrounding land uses with more than two intensity levels. Surrounding agricultural land uses impact biodiversity in three main situations. Firstly, chemical inputs and farming activities affect non-target organisms outside cultivated areas [21, 22]. Secondly, the agricultural matrix influences metapopulation dynamics by influencing species migration [23] or providing a lower quality habitat [24]. Thirdly, various land uses can play different roles during the life cycle of a species [25]; they can provide essential and complementary resources (e.g., nesting and foraging habitats, [26]), provide resources with different availability levels [27], or be dangerous due to a high predation risk, for example [28].

Previous studies that tested biodiversity responses to agricultural intensity found more convex relationships, suggesting that conservation policies would be more effective in locally extensive areas (e.g., [7, 8]). However, these studies did not include empirical testing of the effects of intensity spatial arrangements. Some authors [29, 30] propose that the effect of land use spatial arrangement should be further studied to determine sustainable land use strategies that meet agricultural production and biodiversity conservation goals. If research can identify the shape of the biodiversity/intensity relationship along with the interacting effect of the spatial aggregation of agricultural intensity, conservation policies could be more effective in locally extensive areas surrounded by either low (i.e., aggregated [31, 32]) or high [33, 34] agricultural intensities.

The objective of this study was to test two hypotheses: (1) the relationship between a community of farmland birds and agricultural intensity varies according to species traits, and (2) this relationship is influenced by the spatial aggregation of agricultural intensity. To do this, we used a continuous intensity indicator that incorporates several input categories and was available across a nationwide gradient. We focused on a community of farmland birds and their response to agricultural intensity, and we determined the shape of the relationship between several descriptors of the community and the nationwide intensity gradient. Community composition descriptors included trophic level and degree of specialisation, both which have been shown to be good indicators of habitat disturbance [35]. We used data from the French Breeding Bird Survey (FBBS), a nationwide monitoring program, to compute descriptors of the farmland bird community from 2006 to 2008. Finally, we tested for an interaction effect between agricultural intensity and its spatial aggregation on the bird community descriptors.

## Methods

### Agricultural intensity and its spatial aggregation

**Fig 1:**
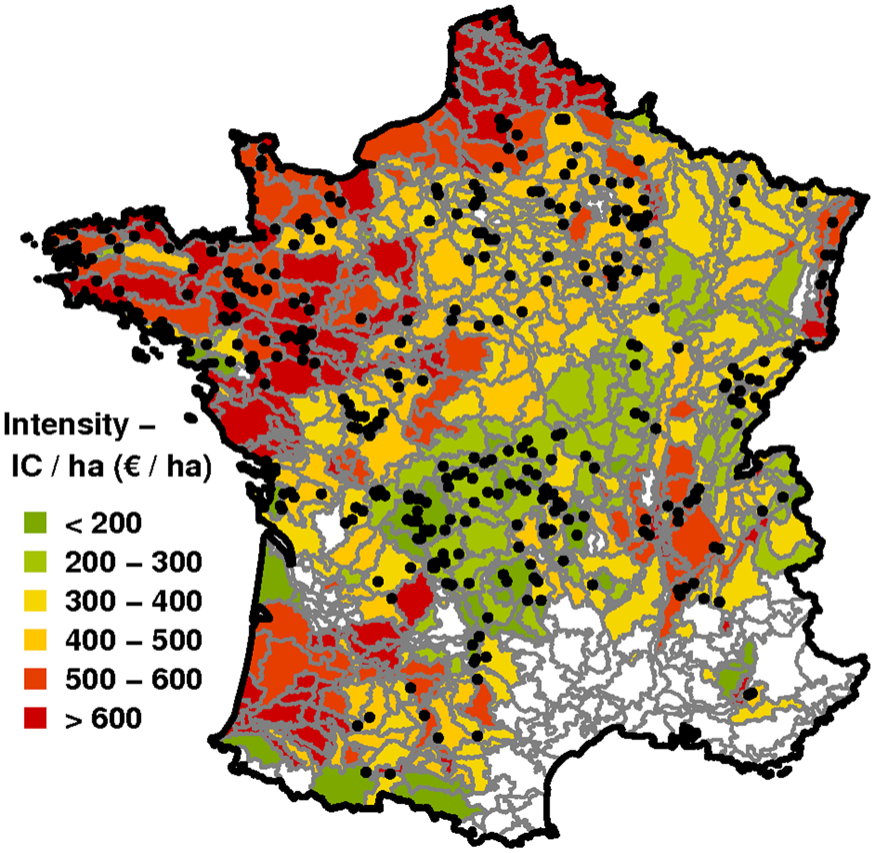
Agricultural intensity value (*Input Cost/ha*, IC/ha) of SARs and FBBS sample sites (black dots) included in our analysis. Sample sites were surveyed between 2006 and 2008 and located in SARs dominated by industrial crops, cereals, bovine dairy, bovine meat, and mixed (crop/bovine) productions. SARs dominated by other production types appear in white. Continuous IC/ha values are shown as six classes, from lowest (green) to highest (red) intensity (see legend). SAR borders appear in grey.

Agricultural intensity is defined as increased utilisation or productivity of land [36] and therefore can be described using either output-oriented (i.e., production) or input-oriented (i.e., utilisation) measures [37, 38]. We used an input-oriented measure of intensity, the Input Cost per hectare (IC/ha) intensity indicator, where IC is expressed in Euros [39]. In the IC/ha ratio, IC is the sum of different categories of input costs, and ha indicates the total utilised agricultural area of a farm. IC categories include fertilizers, feedstuff, pesticides, seeds, fuel, veterinary products, and irrigation water. The IC/ha index was computed at the Small Agricultural Region (SAR) level (Fig. 1) from 2004–2006 data provided by the French Observatory of Rural Development (ODR: http://esrcarto.supagro.inra.fr), a service unit of the French Institute for Agricultural Research that manages agricultural data for research access. In order to overcome year-to-year variation in the price and stock of inputs, the 2006 IC/ha value was an average of years 2004, 2005, and 2006. SARs define consistent units in terms of pedo-climatic conditions, agricultural production systems, and their history [40], which can be important factors influencing the effect of intensity on biodiversity [41].

The use of costs enabled us to combine several categories of inputs of agricultural intensity for different types of agricultural production. Some input categories (e.g., pesticides and fertilizers) have direct negative effects on birds and their habitat, such as toxicity, decreased availability of food resources, and nesting sites [42]. Others (e.g., feed stuffs and seeds) have indirect effects that collectively put global pressure on habitats, such as intensive livestock farms with high feed costs that produce high rates of nitrogen dissipation [43]. We computed IC/ha for five production types industrial crops, cereals, bovine dairy, bovine meat, and mixed (crop/bovine) which together account for 67% of French farms and cover 80% of French agricultural lands. Remaining production types excluded from the IC/ha computation included vegetables of low territorial importance, granivore livestock (poultry and pigs), and wine and orchards where input levels display extremely high values.

To measure the spatial aggregation of the agricultural intensity of any SAR *i* (*AI*_*i*_), we computed the difference between its IC/ha and the mean IC/ha of its contiguous neighbours:

$$\begin{array}{c} AI_{i}=\left|X_{i}-\frac{1}{\sum_{j}w_{i.}}\sum_{j}w_{ij}X_{j}\right| \end{array}$$ (1)

where *X*_*i*_ is the IC/ha value of the SAR *i*, and *w* is the connectivity matrix of all SARs. *w*_*ij*_ = 1 if SARs *i* and *j* are connected, and *w*_*ij*_ = 0 in all other cases. To choose the distance class at which SARs should be connected, we used the Morans I index of spatial autocorrelation [44]. We computed a spatial correlogram of Morans I plotted against the distance connectivity classes. Maximum spatial autocorrelation was reached for contiguous SARs; therefore, only contiguous SARs were considered connected in the connectivity matrix (*w*_*ij*_ = 1). Aggregated SARs had aggregation values *AI* lower than the average value.

### Bird data

Bird data were collected by the French Breeding Bird Survey (FBBS), a standardised monitoring program implemented at the national scale, in which skilled volunteer ornithologists count breeding birds in randomly selected sites each spring [45]. On 2 × 2 km survey sites, observers conduct ten evenly distributed point counts. Point counts are unbounded; observers record every individual bird either heard or seen, along with the distance of contact (<25m, 25–100m, >100m), during a 5-min survey. Surveys are conducted twice each spring.

We calculated the relative abundance of each bird species at each sample site. Because we focused our study on farmland birds, we only included sites with at least five farmland point counts. When sites had more than five farmland point counts, we randomly selected five counts. As each point was surveyed twice per year during the spring, we chose the maximum of the two counts for each species [46]. We then summed the counts at the five points within each square to obtain a yearly local relative abundance of a species per square.

Intensity values were from 2006; therefore, we used bird relative abundance from 2006 to 2008 to account for potential delayed effects of agricultural intensity on bird abundance. Including more years also enabled the smoothing of sampling errors and short-term fluctuations in numbers [47]. The number of surveyed years varied among squares. To avoid certain squares from contributing more than others when testing for the effect of agricultural intensity, we averaged the local relative abundances across years.

The final sample of FBBS sites consisted of 332 sites located in 152 SARs. FBBS sites had to be located in SARs where the total area of the five production types was greater than two-thirds of the total agricultural area. Of the 332 sites, 103 were located in aggregated intensive SARs (i.e., IC/ha more than the national average); 63 were located in non-aggregated intensive SARs; 121 were located in aggregated extensive SARs (i.e., IC/ha less than the national average); and 45 were located in non-aggregated extensive SARs.

We confirmed that there was no bias in the detectability of the different bird species in intensive vs. extensive SARs (following the method of Jiguet et al. 2006 [48], see S1 Appendix).

#### The studied bird community

Both the temporal trends and spatial distributions of bird communities react strongly to agricultural intensity [49, 50]. Among all bird species, farmland birds have been particularly affected by agricultural intensification [45, 50]. We focused on a community of 22 bird species (Table 1) classified as farmland birds by the European Bird Census Council [51]. The community encompassed species nesting on the ground, either in grassland or arable land (e.g., fallows, crops), or in trees or shrubs present in agricultural landscapes. Species found most of their food resources within agricultural lands. We used four variables to describe the bird community. The first index was species richness, which describes the size of the community. The three other indices describe the community composition: community specialisation for farmland over other habitats (Community Specialisation Index, CSI), mean trophic level in the community (Community Trophic Index, CTI), and community specialisation for grassland over other farmland habitats (Community Specialisation for grassland Index, CSIg).

**Table 1:** Farmland bird community: species, habitat specialization index, trophic index, and grassland specialization index.

Species	Specialisation index (CSI)	Trophic index (CTI)	Grassland specialisation index (CSIg)
*Perdix perdix*	1.31	1.10	1.25
*Motacilla flava*	1.19	2.00	1.33
*Miliaria calandra*	1.08	1.28	1.56
*Vanellus vanellus*	1.55	1.90	1.56
*Carduelis chloris*	0.86	1.05	1.58
*Coturnix coturnix*	1.21	1.22	1.59
*Alauda arvensis*	1.13	1.25	1.60
*Carduelis carduelis*	0.67	1.05	1.66
*Alectoris rufa*	0.69	1.10	1.84
*Carduelis cannabina*	0.62	1.05	1.85
*Corvus frugilegus*	0.92	1.63	1.94
*Anthus pratensis*	1.33	1.75	2.00
*Sylvia communis*	0.63	1.60	2.04
*Falco tinnunculus*	0.48	2.85	2.12
*Emberiza citrinella*	0.54	1.30	2.26
*Saxicola torquatus*	0.66	2.00	2.29
*Emberiza cirlus*	0.39	1.30	2.37
*Buteo buteo*	0.39	2.90	2.42
*Saxicola rubetra*	1.23	2.00	2.44
*Upupa epops*	0.29	2.00	2.53
*Lanius collurio*	0.87	2.15	2.58
*Lullula arborea*	0.58	1.50	2.61

The CSI was computed as:

$$\begin{array}{c} CSI=\sum_{i=1}^{n}\frac{N_{i}}{N_{tot}}*SSI_{i} \end{array}$$ (2)

where *SSI*_*i*_ is the specialisation index of each species, *i*, weighted by its abundance, *N*_*i*_, and divided by the summed abundances of all 22 species, *N*_*tot*_. SSI indicates whether a species is only associated with farmlands or can be found in other habitats. Similar to Julliard et al. (2006) [52], we set SSI equivalent to the species density coefficient of variation (standard deviation divided by the mean, which is statistically independent of the average species density) for seven habitat classes: forest, heath/scrub, marshland, farmland, urban settlement, wetland/aquatic environment, and rocks. These habitat classes were recorded with bird abundances at each FBBS site. We computed SSIs for all FBBS sites from 2006 to 2008. At the community level, CSI is high when the community is dominated by highly specialised farmland species.

The CTI describes a community functional composition. Similar to the CSI, it is also computed as a summation of indices (species trophic indices, *STI*_*i*_) weighted by abundances:

$$\begin{array}{c} CTI=\sum_{i=1}^{n}\frac{N_{i}}{N_{tot}}*STI_{i} \end{array}$$ (3)

where *STI*_*i*_ is the proportion of seeds/plants, invertebrates, and vertebrates in the species diet, weighted by 1, 2, and 3, respectively [45]. The proportions of these three elements in the diet were previously recorded in the Bird of the Western Paleartic interactive [53]. At the community level, the CTI is high when invertebrate-eating species are dominant in the community and low when granivore species are dominant in the community.

We further calculated the CSIg to determine whether the community was dominated by species whose main habitat was grassland or arable land [54]. Similar to the other indices, the CSIg was computed as:

$$\begin{array}{c} CSIg=\sum_{i=1}^{n}\frac{N_{i}}{N_{tot}}*SSIg_{i} \end{array}$$ (4)

where SSIg is the weighted mean of species abundance among four sub-habitats of the farmland habitat: unimproved grasslands, improved grasslands, mixed grasslands/arable lands, and arable lands. Weighting coefficients were 4, 3, 2, and 1, respectively. All farmland FBBS sites surveyed between 2006 and 2008 were included in this computation.

The habitat specialisation of a particular species often varies among geographical regions (e.g., differences between France and Sweden, [55])). It is thus interesting to use data and habitat association measures to compute the average habitat specialisation over a large area like France. Reif et al. (2010) [56] showed that specialisation indices obtained from expert opinion, breeding bird survey datasets compiled with similar methods as those used here, or other statistical approaches lead to similar or highly correlated classifications. The SSI has been widely used in studies assessing the effects of land use and intensity [11, 35, 52, 57]. Although developed more recently, the SSIg uses the same principles as the SSI and has been used to study the effect of farmland heterogeneity on birds [54].

### Climate and land use data

On the large geographical gradient of our study (i.e., national), agricultural intensity is not the only factor influencing bird populations; climate and land use are also likely to have an effect. Therefore, climate variables included mean temperature and annual precipitation. Data were available at the SAR level from Mto-France, the French meteorological institute, through the ODR. We averaged data from three years (2006 to 2008), consistent with the bird data. Using CORINE land cover raster data, we computed variables related to land use at the scale of bird sample sites. Variables were the proportions of 15 CORINE land cover categories in the sample site, including categories reflecting landscape heterogeneity (e.g., complex cultivation patterns, agricultural lands with significant areas of natural vegetation). To further describe landscape heterogeneity, we computed a Shannon index of land cover diversity. As intensity partially correlated with a land use gradient from grassland to arable land (S2 Appendix), we also included a variable computed as the ratio between arable and grassland area (arable area divided by the summation of arable and grassland areas) at the SAR level (i.e., same level as the intensity data). We tested for an interaction effect between intensity and the arable/grassland ratio in separate Generalized Linear Models (GAMs). As the interaction effect was not significant for any response variable, we did not include it in the final analyses. Finally, we included an altitude variable computed as the mean altitude in the bird sample sites from Instut Gographique National (IGN, French National Institute for Geography) data. The full list of explanatory variables is detailed in Table 2.

**Table 2:** List of all explanatory variables.

Category	Variables
Agricultural intensity	IC/ha intensity indicator, intensity aggregation
Proportion of CLC categories	Non-irrigated arable land, Vineyards, Fruit trees and berry plantations, Pastures, Complex cultivation patterns, Land principally occupied by agriculture, with significant areas of natural vegetation, Broad-leaved forest, Coniferous forest, Mixed forest, Natural grasslands, Moors and heathland, Transitional woodland-shrub, Artificial surfaces, Wetlands, Water bodies
Other land use variables	Shannon diversity of CLC categories, arable land/grassland ratio
Climate variables	Mean temperature, Annual precipitation
Altitude	Altitude

CLC = CORINE land cover

### Statistical analysis

#### Non-linear effect of agricultural intensity on the bird community

We used GAMs to test the intensity effect on the size and composition of the bird community. Responses to agricultural intensity may not always be linear; hence the use of GAMs, which can accommodate more complex patterns [58]. The general formula of these GAMs was:

$$\begin{array}{c} \begin{array}{c} Bird\;community\;descriptor∼s(IC/ha)+altitude+\\ land\;use\;variables+climate\;variables \end{array} \end{array}$$ (5)

We used all four bird community descriptors (species richness, CSI, CTI, and CSIg) as response variables. The IC/ha intensity indicator was a continuous explanatory variable that was integrated into the GAMs as a spline function with two degrees of freedom (*s*()) to minimise the AIC and general cross-validation criteria for all models. Additional explanatory variables included altitude and the previously described set of land use and climate variables. Due to the large number of explanatory variables, we used a backward stepwise model selection procedure. Starting from the maximal model containing all predictors, this procedure performs iterative variable selection based on AIC criterion, which leads to a minimally adequate model including only the predictors with the highest explanatory power. The minimally adequate model is the model that produces the least unexplained variation while retaining the minimal number of predictors according to the parsimony principle [59]. The results present the performance of these minimal adequate models. We used a leave-one-out cross-validation procedure to assess the predictive ability of the models. For each model, we computed the mean cross-validation error, expressed as a percentage of the observed value [60, 61]. We tested all response variables for normal distributions and homoscedasticity of residuals. We also checked that there was no spatial autocorrelation in the model residuals between adjacent SARs. To do this, we used the Morans I index [44, 62] and assessed its significance using a bootstrap procedure (i.e., the *spdep* package in R statistical software). GAM models were computed with the *mgcv* package in R statistical software.

#### Interaction effect between agricultural intensity and its spatial aggregation on the bird community

SARs were divided into aggregated and non-aggregated groups as previously described to test for an interaction effect between agricultural intensity and its spatial aggregation. Because of this sample division, the use of non-linear models (i.e., GAMs) would have led to over-fitting. Therefore, we used generalized linear models (GLMs) to test for interaction effects on the four bird community descriptors. The general formula was:

$$\begin{array}{c} \begin{array}{c} Bird\;community\;descriptor∼IC/ha*aggregation+arable/grassland\;ratio+\\ +altitude+land\;use\;variables+climate\;variables \end{array} \end{array}$$ (6)

Similar to the GAM models (Eq. 5), we used all four bird community descriptors as response variables; we included altitude, land use, and climate as explanatory variables; and we performed the same model selection procedure. We tested for an interaction effect (* symbol) between intensity (IC/ha indicator) and its spatial aggregation. The IC/ha was a continuous explanatory variable, whereas its aggregation was a binary factor that was equal to 0 in non-aggregated SARs and 1 in aggregated SARs. Therefore, the models computed differences in the intercept and slope of the effect of intensity in aggregated SARs compared with non-aggregated SARs. Because SAR spatial units are heterogeneous in size, we examined whether this had an influence on the effect of intensity and its spatial aggregation on the bird community by testing for an interaction effect among intensity, aggregation, and SAR size. As this effect was not significant for any of the bird response variables, we did not keep it in the final models.

## Results

### Non-linear effect of agricultural intensity on the bird community

**Fig 2:**
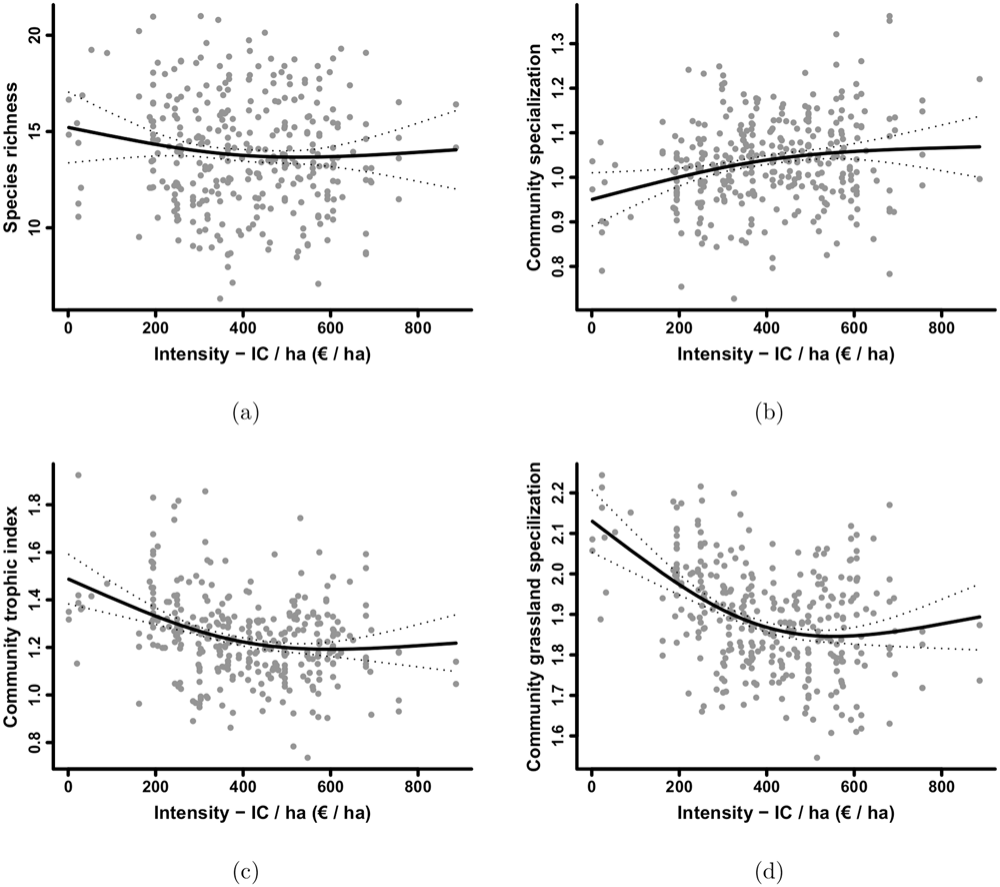
Effects of the *Input Cost/ha* (IC/ha) intensity indicator on size and composition of the bird community. Effect on (a) species richness, (b) community specialisation index, (c) community trophic level, and (d) grassland specialisation index of the bird community. Black curves: responses to the IC/ha intensity indicator as predicted by the GAM, and plotted with 95% confidence intervals (dotted lines) and partial residuals (grey points).

Agricultural intensity had a stronger effect on community composition than on species richness (Fig. 2 and Table 3). The relationship between IC/ha intensity and community species richness was weakly significant (*p* = 0.045, Fig. 2a). However, the three community composition descriptors were significantly affected by IC/ha intensity, indicating that the community was comprised of both intensity “loser” and “winner” species. Similarly, the predictive ability of the model was low for species richness (cross-validation error = 31%) but high for the community composition descriptors (cross-validation error = 11%, 9%, and 5% for CSI, CTI and CSIg, respectively).

**Table 3:** Performance summary of the GAMs computing the effects of the *Input Cost / ha* (IC/ha) intensity indicator on the four bird community descriptors.

	Estimate	Standard error	Test statistic	p-value
**Species** richness				
Intercept	14.017	1.058	13.245	< 0.001 ***
IC/ha			1.015	0.364
Mean temperature	-0.765	0.155	-4.943	< 0.001 ***
Annual precipitation	-7.992	1.359	-5.882	< 0.001 ***
Altitude	-0.005	0.002	-3.401	0.001 **
Non-irrigated arable land	2.29	0.7	3.272	0.001 **
Pastures	3.852	0.908	4.241	< 0.001 ***
Moors and heathland	24.855	10.405	2.389	0.017 *
**CSI**				
Intercept	0.754	0.04	18.928	< 0.001 ***
IC/ha			6.055	0.003 **
Arable/grassland ratio	0.136	0.047	2.889	0.004 **
Mean temperature	-0.013	0.003	-4.071	< 0.001 ***
Annual precipitation	-0.241	0.045	-5.3	< 0.001 ***
Non-irrigated arable land	0.279	0.023	11.963	< 0.001 ***
Natural grasslands	0.928	0.175	5.304	< 0.001 ***
Artificial surfaces	0.248	0.062	3.974	< 0.001 ***
**CTI**				
Intercept	1.262	0.07	18.131	< 0.001 ***
IC/ha			12.23	< 0.001 ***
Mean temperature	-0.017	0.009	-1.819	0.07
Annual precipitation	0.161	0.082	1.962	0.051
Altitude	0	0	-2.077	0.039 *
Non-irrigated arable land	0.144	0.06	2.397	0.017 *
Pastures	0.34	0.066	5.123	< 0.001 ***
Complex cultivation patterns	0.371	0.078	4.729	< 0.001 ***
Agriculture with natural vegetation	0.455	0.146	3.113	0.002 **
Mixed forest	0.244	0.179	1.364	0.174
Transitional woodland-shrub	0.541	0.346	1.565	0.119
**CSIg**				
Intercept	1.63	0.034	47.946	< 0.001 ***
IC/ha			24.09	< 0.001 ***
Annual precipitation	0.192	0.055	3.487	0.001 **
Vineyards	0.39	0.158	2.469	0.014 *
Pastures	0.405	0.032	12.512	< 0.001 ***
Complex cultivation patterns	0.305	0.038	8.047	< 0.001 ***
Agriculture with natural vegetation	0.476	0.1	4.741	< 0.001 ***
Broad-leaved forest	0.25	0.052	4.856	< 0.001 ***
Mixed forest	0.471	0.118	3.99	< 0.001 ***
Natural grasslands	-0.505	0.213	-2.368	0.018 *
Transitional woodland-shrub	0.694	0.241	2.879	0.004 **

n = 332 points for all models; test statistic = *F* for IC/ha and *t* for other variables; CSI: community specialisation index, CTI: community trophic index, CSIg = grassland specialisation index of the community;

* p-value < 0.05,

** p-value < 0.01,

*** p-value < 0.001

Agricultural intensity had a positive effect on the CSI (Fig. 2b) and a negative effect on both the CTI and CSIg of the community (Fig. 2c and 2d). That is, loser species were invertebrate-eating birds (i.e., high trophic level) with moderate farmland specialisation and preference for grassland habitats, and they dominated the bird communities of extensive SARs. Conversely, winner species were seed-eating birds (i.e., low trophic level) with high farmland specialisation and preference for arable habitats, and they replaced loser species in more intensive SARs. A weak negative effect of intensity on species richness indicated that the bird community included slightly more loser species than winner species.

The effect of intensity on all community composition descriptors was non-linear and stronger at low intensities. The shape of the relationship between bird community composition descriptors and agricultural intensity was convex for negative relationships and concave for positive relationships. For all descriptors, the intensity effect was strong at low intensities and attenuated (becoming almost null) at higher intensity levels where IC/ha values > 400 Euros/ha (i.e., approximately the national mean IC/ha value, 405.1 Euros/ha).

### Interaction effect between agricultural intensity and its spatial aggregation on the bird community

**Fig 3:**
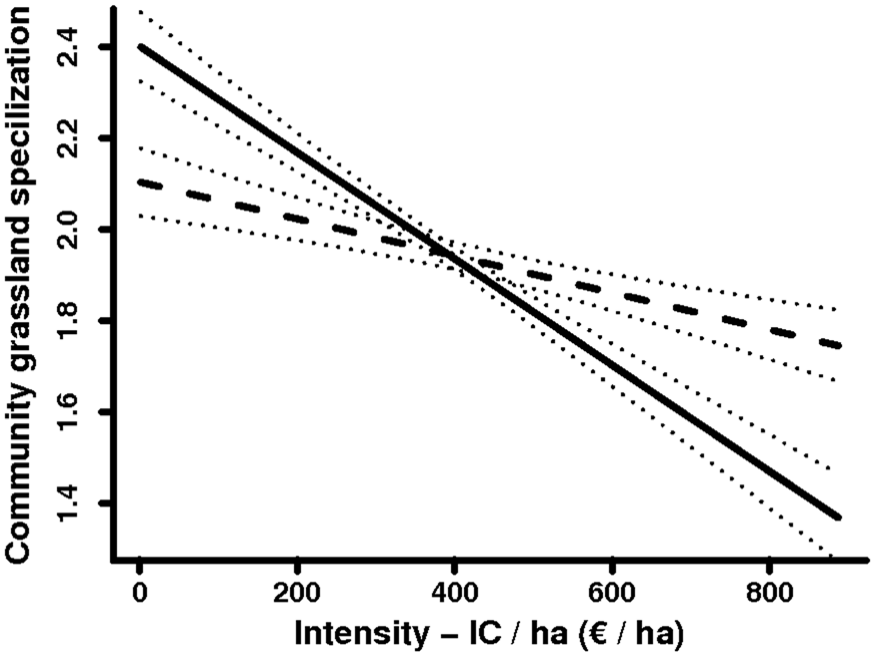
Interactions between agricultural intensity (*Input Cost/ha*, IC/ha) and intensity aggregation on the grassland specialization index of the bird community. The two curves represent the effect in SARs either aggregated (solid line) or non-aggregated (dashed line) with neighbours of similar intensity. Dotted lines = 95% confidence intervals.

For the CSIg of the bird community, the effect of agricultural intensity was stronger when intensity was spatially aggregated (i.e., in SARs with contiguous neighbours of similar intensity; Fig. 3 and Table 4). The interaction effect between intensity and its spatial aggregation was highly significant (with significant differences for both intercepts and slopes; Table 4), and the model had good predictive ability (cross-validation error = 5%). The CSIg of the bird community was significantly higher in extensive SARs when they were aggregated but significantly higher in intensive SARs when they were non-aggregated. Grassland birds (i.e., with high SSIg values; Table 1) were more abundant in extensive SARs when aggregated and in intensive SARs when non-aggregated. Conversely, arable birds (i.e., with low SSIg values) were more abundant in extensive SARs when non-aggregated and in intensive SARs when aggregated. Therefore, aggregation had a positive effect within the species-favourable range of intensity and a negative effect outside this range. No significant interaction effects were observed for the other community descriptors (Table 4).

**Table 4:** Performance summary of the GLMs computing the interaction effect between agricultural intentity (*Input Cost / ha* indicator, IC/ha) and its spatial aggregation on the grassland specialisation index of the community (CSIg).

	Estimate	Standard error	*t*	p-value
**CSIg**				
Intercept	1.779	0.066	26.964	< 0.001 ***
IC/ha	-1.165e-03	9.264e-05	-12.57	< 0.001 ***
Aggregation	-0.297	0.053	-5.607	< 0.001 ***
IC/ha*Aggregation	7.602e-04	1.218e-04	6.241	< 0.001 ***
Mean temperature	0.012	0.004	2.806	0.005 **
Annual precipitation	0.269	0.061	4.41	< 0.001 ***
Vineyards	0.392	0.159	2.459	0.014 *
Pastures	0.398	0.039	10.299	< 0.001 ***
Complex cultivation patterns	0.268	0.041	6.518	< 0.001 ***
Agriculture with natural vegetation	0.443	0.104	4.263	< 0.001 ***
Broad-leaved forest	0.273	0.053	5.136	< 0.001 ***
Mixed forest	0.431	0.123	3.498	0.001 **
Natural grasslands	-0.457	0.222	-2.061	0.04 *
Transitional woodland-shrub	0.712	0.245	2.906	0.004 **

“Aggregation” is the difference in intercept and “IC/ha*Aggregation” is the difference in slope in non aggregated SARs compared to aggregated SARs. n = 332 points for all models; CSI: community specialisation index, CTI: community trophic index, CSIg = grassland specialisation index of the community.

## Discussion

### Strengths and limitations of the IC/ha intensity indicator

The IC/ha indicator provides a continuous value that combines several categories of inputs that have an effect on biodiversity. For some categories, the effect on biodiversity and its habitat is direct (e.g., for fertilizers, pesticides, veterinary products, and irrigation) [63–65]. For other categories, this effect is indirect (e.g., higher feed costs are associated with higher livestock densities and nitrogen dissipation; fuel inputs reflect farming activities that create disturbance; seeds are usually treated with crop protection products and sold along pesticides in a technological package) [43, 66]. The combination of several input categories makes the IC/ha indicator relevant for both livestock and crop productions. These productions cover nearly 80% of French agricultural lands; therefore, the IC/ha indicator can describe a wide-range, nationwide gradient of agricultural intensity. The IC/ha can also discriminate intensity levels both within and across production systems; interestingly, it shows that livestock and crop production systems alternate along the intensity gradient [39]. The IC/ha is based on data from the Farm Accountancy Data Network, a survey that follows the same methodology in the 27 countries of the European Union, and it does not rely on expert opinion. Therefore, it provides a highly reproducible measure of intensity.

A large number of studies at farm scale focused on a single input category, most often nitrogen [7, 67], to describe intensity. These studies can isolate the effect of one input category on biodiversity but cannot account for substitution effects [68]. For instance, two systems with similar nitrogen input levels can have very different overall intensity values because the levels of other input categories are different [39]. In contrast, the IC/ha indicator accounts for several input categories but does not capture their different effects on biodiversity (e.g., pesticides being more detrimental than irrigation). Studies that focus on a single input category could be complementary to our approach, as they could identify the categories with the most adverse effects on biodiversity. Ideally, the IC/ha indicator should be dissociated to examine the relative impact of the different input categories on biodiversity; however, this was not possible with the present method [39] and data availability. Indeed, data availability is an important limiting factor for studies addressing the spatial distribution of agricultural intensity at a large scale, which may explain why the dichotomous (e.g., organic vs. conventional farming [32, 69] or high nature value areas [70, 71]) or discrete [72, 73] description of intensity prevails in such studies. Although computed at lower resolution, the IC/ha is a continuous indicator, which is an improvement over discrete intensity metrics.

In order to combine different categories of inputs into the IC/ha indicator, cost was used as a common unit. The use of cost results in an absolute intensity value, unlike the separate normalisation of different categories that leads to a relative value [74], or the use of a score which incorporates arbitrary computational choices [75]. The use of cost also carries limitations. The proportionality between costs and amounts can be biased by fluctuations in input prices or stocks. In order to partially overcome this limitation, the 2006 IC/ha value was averaged over the 3 previous years. Comparison of the IC/ha indicator with other intensity indicators showed good consistency. Nearly all (96%) of the low input SARs (quantile 0.2 of IC/ha, IC/ha < 300 Euros/ha) included municipalities with a high nature value status [75]. There was a strong correlation between the IC/ha and stocking rate, which has been widely used to describe the intensity of livestock production (S3 Appendix). Both output-oriented measures (e.g., yield) and input-oriented measures can be used to describe agricultural intensity [76]. Biodiversity is more directly impacted by the intensity of management practices such as input use. Yield correlates with management intensity but also depends on pedo-climatic conditions. Consistent with this prediction, there was a significant correlation between the IC/ha and yield for both crop and livestock production (S3 Appendix).

### Underlying mechanisms of the effect of agricultural intensity and its spatial aggregation

Agricultural intensity had a stronger effect on bird community composition than on species richness. Doxa et al. (2010) [57] found no significant difference in the taxonomic diversity of a French bird community in intensive vs. extensive areas, but its average specialisation index was influenced. These effects on community composition imply that “winner” replace “loser” species as agricultural intensity increases. The presence of such winner and loser species has already been shown in the context of habitat disturbance [35] and agricultural yield [8]. It pleads for use of several community composition indicators when policy makers address the effects of intensity and determine priority species and actions.

Although agricultural intensity was partially correlated to land use (S2 Appendix), Teillard et al. (2012) [39] showed that crop and livestock systems alternated along the IC/ha gradient. In our study, winner species benefited from more intensive areas, which has also been documented in Europe [77, 78]. This does not mean that winner species directly benefit from higher levels of inputs; on the large intensity gradient that we describe, the IC/ha indicator is very likely to be correlated with other intensity components. For instance, in the most extensive landscapes, the lack of nearby crop fields limits foraging opportunities for seed-eating birds in winter and influences local breeding densities [79, 80]. Intensive and homogeneous landscapes benefit specialists of open field habitats [11]. The input level component of the IC/ha may explain why the positive response of winner species was attenuated and why species richness was slightly decreased at high intensities. It reflects the negative effect of high input levels on biodiversity that has been widely documented in the literature (see [81] and review in [42]). Phalan et al. (2011) [8] also found that more species displayed concave positive responses to yield than convex responses. Here, we averaged intensities at the SAR scale and could not examine the effects of very high intensities that can be found at lower scales (e.g., one to several farms) that may be detrimental even to winner species.

Most local studies that compare farmland specialists to habitat generalists show specialists to be the intensity loser species [10, 11]. We found grassland specialists to be loser species, with high sensitivity to intensity (i.e., convex negative response); however, their degree of specialiszation in farmlands was lower than that of arable specialists, which that seemed better adapted to higher agricultural intensity. As a result, our community specialiszation index was higher in intensive areas. Even with small levels of intensification, the habitat quickly became unsuitable for specialiszed grassland species. This result highlights the importance offor hosting a unique pool of specialiszed species in extensive European grasslands [14].

The farmland bird community was significantly impacted by intensity aggregation (i.e., by the intensity of contiguous SARs). Annual effects are not likely to be dominant at this scale as summer territory size [82] and winter foraging movements [83] are generally smaller than a SAR. Surrounding SARs are likely to influence the observed community composition through an impact on their longer-term metapopulation dynamics. Evidence of the metapopulation dynamics of birds in farmland habitats already exists [19, 84]. Moreover, the scale at which surrounding habitats influence the stability of bird metacommunities matches the scale that we addressed in our study [20]. Other studies have addressed the interacting impact of intensity with properties of the surrounding area. Most of these studies took place at smaller scales, in which the surrounding landscape was within a few hundred meters radius [85–87]. These previous studies revealed significant interactions; local management improvement yielded higher biodiversity benefits when the surrounding landscapes were simple (i.e., intensive). We found that intensity changes had stronger effects in extensive SARs that were aggregated with other extensive surrounding neighbours. This result is in line with other large scale studies that found that agri-environmental schemes (AESs) produce greater biodiversity benefits in more extensive countries [88, 89] and in small regions (10 × 10 km) with already high AES concentrations [90]. Therefore, the effects of interactions between local intensity and the intensity of surrounding areas differ between landscape and larger scales.

### The significant effect of the spatial aggregation of agricultural intensity: implications for conservation

The non-linear relationship between agricultural intensity and community composition descriptors was stronger in the lower intensity range. One consequence is that winner species already dominate the bird community at mean intensity levels; therefore, there is an imbalance in favour of these species in France. The non-linear relationship also supports the hypothesis that policies promoting extensive practices would elicit higher benefits to loser species in extensively farmed than in intensively farmed agricultural regions [6, 31]. In the context of our study, one option would be for policies to aim at preserving very extensively managed areas. Remaining areas could possibly favour species that benefit from agricultural intensity, if that were the case (i.e., our results do not support positive effects on species at a higher intensity than the maximum of our gradient).

In Europe, current AESs already tend to focus on extensive areas (i.e., “less-favoured areas”), where, according to our results, they are expected to be the most effective. AES effectiveness, however, is currently questionable [4, 5]. We found that the effect of agricultural intensity on biodiversity was stronger in areas where intensity was spatially aggregated. This result could partially explain the low effectiveness of AESs when uptake rate is spatially diffuse [6, 90]. Conversely, targeting and concentrating policies in areas with spatial aggregation of intensity could be an opportunity for improving their effectiveness [91, 92]. Our results support the general argument that policy measures targeting areas where extensive farmlands are aggregated will yield the greatest environmental benefit [6, 31]. However, they also show that agricultural intensity is not the only factor influencing the farmland bird community; land use and landscape variables also explained a large part of the variation. This means that policy measures focusing solely on intensity would have limited effect. Heterogeneity at the landscape level has been shown to have a strong influence on farmland biodiversity and, furthermore, the effects of landscape heterogeneity can interact with farming intensity [34, 86]. Disentangling these effects was not possible in our study due to the nature of our intensity data (i.e., large scale and combining several categories of inputs). Policy measures should not be excluded from intensive areas, where their positive influence has been emphasised for endangered species [93, 94] and at the landscape scale [33].

In the land sparing/land sharing model [95], the relative benefit of these two land use allocation strategies depends on the shape of the trade-off between biodiversity and yield. Several authors have suggested that including the effect of intensity spatial allocation would improve this model [19, 29, 30]. Our results confirm this improvement. Indeed, the land sparing strategy corresponds to a high level of intensity aggregation, as the two intensity extremes are spatially segregated: low intensities to fulfil biodiversity objectives vs. high intensities to fulfil production objectives. We show that such aggregation on a large spatial scale can impact biodiversity. On such large scale, agricultural intensity is already spatially structured in several countries (e.g., organic farming in the United Kingdom [32]; high nature value areas in France [75]; intensive vs. extensive areas in France [39]). If the effects of spatial aggregation are not carefully assessed, bias could occur in the sample used to compute the biodiversity/production trade-offs and thus affect the conclusions drawn from their shapes.

Improving the effectiveness of conservation policy on the scale of countries or all of Europe is crucial for reversing biodiversity loss. On such large scales, our results suggest that targeting conservation efforts in areas of aggregated extensive agriculture could be a way to achieve this improvement. The exponential decline of loser species with increasing intensity supports the important role of extensively managed habitats for biodiversity in Europe (as already highlighted by [14, 96]). The value of natural habitats should be further assessed to conclude which habitats gain priority. Both extensive and natural habitats may be necessary. We show that the spatial aggregation of agricultural intensity can influence biodiversity. Consideration of the spatial arrangement of intensity will be important in models seeking to inform sustainable land use allocation strategies, such as the land sparing/sharing framework.

## Supporting Information

S1 Appendix Detectability of bird species in extensive vs. intensive Small Agricultural Regions. (PDF)

S2 Appendix Correlation between the agricultural intensity gradient and the land use gradient. (PDF)

S3 Appendix Correlation between the IC/ha indicator and other intensity metrics. (PDF)
