# Supplementary material for: The Response of Farmland Bird Communities to Agricultural Intensity as Influenced by Its Spatial Aggregation
Source: PLoS One. 2015 Mar 23;10(3):e0119674. doi: 10.1371/journal.pone.0119674 (PMC4370717; doi:10.1371/journal.pone.0119674)
Supplement: S1 Appendix — (PDF) [file pone.0119674.s001.pdf]

Appendix S1 – Detectability of the bird species in extensive vs. intensive Small Agricultural Regions

We computed a matrix of the presence / absence of each species, at each point count in order to compare their detection probability between extensive SARs and intensive SARs (first and last quartiles of IC/ha values, respectively). We used the model M(h) which stipulates that the detection probability can vary between sites (between rows of the matrix), and the associated jackknife estimator [1]. Detection probabilities were obtained by running the program MARK [2]. No important differences were found between the detection probabilities of the species in extensive vs. intensive SARs (Fig. S1).

Moreover, we checked for a pattern in the detection probabilities of the different species between extensive and intensive SARs, which could result in wrong inference regarding the effect of intensity. We classified species in three groups based on their SSIg values. The eight species with the highest SSIg values were classified as grassland species, the eight species with lowest SSIg values were classified as arable species, and remaining species were classified as mixed habitat species (color legend in Fig. S1). There was no pattern in the detection probabilities of grassland, arable and mixed habitat species (Fig. S1).

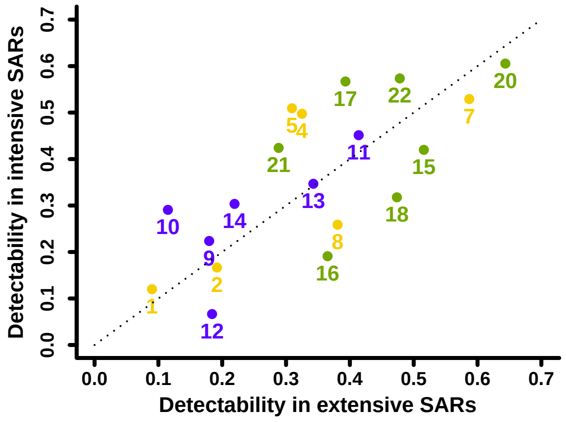

	Species	SSIg
1	*Perdix perdix*	1.25
2	*Motacilla flava*	1.33
3	*Vanellus vanellus*	1.56
4	*Miliaria calandra*	1.56
5	*Carduelis chloris*	1.58
6	*Coturnix coturnix*	1.59
7	*Alauda arvensis*	1.6
8	*Carduelis carduelis*	1.66
9	*Alectoris rufa*	1.84
10	*Carduelis cannabina*	1.85
11	*Corvus frugilegus*	1.94
12	*Anthus pratensis*	2
13	*Sylvia communis*	2.04
14	*Falco tinnunculus*	2.12
15	*Emberiza citrinella*	2.26
16	*Saxicola torquatus*	2.29
17	*Emberiza cirlus*	2.37
18	*Buteo buteo*	2.42
19	*Saxicola rubetra*	2.44
20	*Upupa epops*	2.53
21	*Lanius collurio*	2.58
22	*Lullula arborea*	2.61

Figure S1. Detection probability of the 22 species in extensive vs. intensive Small Agricultural Regions (SARs) (first and last quartile of intensity value, respectively). Green = grassland species, yellow = arable species, blue = mixed habitat species. For three species, samples were too low to compute detectability in one of the two SAR groups: *Saxicola rubetra*, *Coturnix coturnix* and *Vanellus vanellus*. Dotted line: y = x

References

1. Jiguet F, Julliard R, Thomas CD, Dehorter O, Newson SE, et al. (2006) Thermal range predicts bird population resilience to extreme high temperatures. Ecology letters 9: 1321–30.
2. White GC, Burnham KP (1999) Program MARK: survival estimation from populations of marked animals. Bird Study 46: S120–S139.
