# Supplementary material for: The Response of Farmland Bird Communities to Agricultural Intensity as Influenced by Its Spatial Aggregation
Source: PLoS One. 2015 Mar 23;10(3):e0119674. doi: 10.1371/journal.pone.0119674 (PMC4370717; doi:10.1371/journal.pone.0119674)
Supplement: S2 Appendix — (PDF) [file pone.0119674.s002.pdf]

Appendix S2 – Correlation between the agricultural intensity gradient and the land use gradient.

There was a significant correlation between the intensity of the small agricultural regions and (*Input Cost/ha, "IC/ha"* intensity indicator) and their land uses (Fig. S2, $p - value < 0.001$). This correlation was not perfect ($r^2 = 0.29$) because the there was differences in input levels within the land uses (e.g., between two small agricultural regions dominated by arable lands) besides differences between land uses (e.g., between a grassland dominated and an arable land dominated small agricultural region).

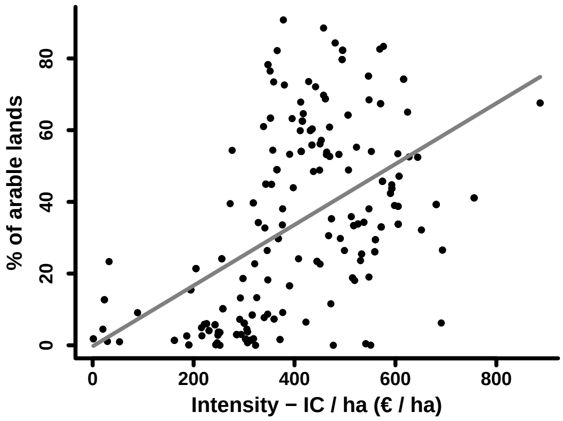

Figure S2. Correlation between the gradient of agricultural intensity (*Input Cost/ha, "IC/ha"* intensity indicator) and a land use gradient (% of arable lands). The regression curve is shown in black.
