# Supplementary material for: The Response of Farmland Bird Communities to Agricultural Intensity as Influenced by Its Spatial Aggregation
Source: PLoS One. 2015 Mar 23;10(3):e0119674. doi: 10.1371/journal.pone.0119674 (PMC4370717; doi:10.1371/journal.pone.0119674)
Supplement: S3 Appendix — (PDF) [file pone.0119674.s003.pdf]

Appendix S3 – Correlation between the IC/ha indicator and other intensity metrics

The Farm Accountancy Data Network (FADN) dataset was used to compute the correlation between the IC/ha indicators and other metrics that have been used in the literature to describe agricultural intensity (Table S3). The FADN contains a very broad set of variables at the individual farm level. It provides a limited sample of farms surveyed on a yearly basis (n = 7361 farms in 2006, ≈ 2% of French professional farms). The sample is designed to give statistical representativeness at the NUTS 2 scale for all the main production type. Correlations were computed by production types.

Table S3. Summary of the quadratic regressions between the IC/ha intensity indicator and two other intensity-related variables (yield and stocking rate), for different types of production.

			IC/ha			IC/ha^2		
	d.f.	r^2	Estimate	t	p-value	Estimate	t	p-value
**IC/ha correlation with yield**								
Crop and mixed production	2350	0.24	9.354e-04	20.774	$< 0.001 ***$	-4.296e-07	-13.944	$< 0.001 ***$
Livestock production	1515	0.39	4.048e-04	14.321	$< 0.001 ***$	-7.099e-08	-3.177	$< 0.001 ***$
**IC/ha correlation with stocking rate**								
Dairy production	947	0.37	2.212e-03	14.826	$< 0.001 ***$	-8.116e-07	-7.588	$< 0.001 ***$
Meat production	587	0.35	1.984e-03	7.651	$< 0.001 ***$	-3.727e-07	-1.249	0.212

Yield was calculated as the ratio between kg of product and ha of utilized agricultural area, stocking rate was calculated as the ration between livestock units and ha of main fodder area. d.f. = degrees of freedom. * $p - value < 0.05$, ** $p - value < 0.01$, *** $p - value < 0.001$.
